# Canine pseudopregnancy: an evaluation of prevalence and current treatment protocols in the UK

**DOI:** 10.1186/s12917-018-1493-1

**Published:** 2018-05-24

**Authors:** Amanda L. Root, Tim D. Parkin, Pippa Hutchison, Caroline Warnes, Philippa S. Yam

**Affiliations:** 1 School of Veterinary Medicine, College of Medicine, Veterinary and Life Sciences, University of Glasgow, 464 Bearsden Road, Glasgow, G61 1QH Scotland; 2 Good Companions, 3 The Hill, Bourton, Swindon, SN6 8JA England

**Keywords:** Aggression - behaviour, Bitch - dog, Cabergoline, Epidemiology, Pseudopregnancy, Pseudocyesis, Questionnaire

## Abstract

**Background:**

There is a dearth of literature on pseudopregnancy in the bitch, with only a few treatment-based studies published since the 1990s. Pseudopregnancy may be under-recognised in bitches and may account for a proportion of behavioural cases seen in veterinary practices including aggression. Little is known about commonly used treatments for overtly pseudopregnant bitches and it is possible that current regimes may not be prescribed for a sufficient duration to control any clinical signs including, physical and behavioural changes. To investigate current trends in diagnosis and treatment of canine pseudopregnancy, a postal survey was sent to 2000 randomly selected veterinary surgeons in UK veterinary practices. The questionnaire queried how often vets recognise cases of pseudopregnancy in spayed and entire bitches, which physical or behavioural signs are commonly recognised for diagnosis, and which management or treatment protocols are used.

**Results:**

The response rate was 19.8% (397/2000). Ninety-six percent of veterinary surgeons reported seeing pseudopregnant bitches showing behavioural changes without any physical changes within the last 12 months. Of those behavioural changes, collecting and mothering objects was the most frequently reported behavioural sign (96%). Ninety-seven percent of vets had seen aggression in pseudopregnant bitches. Nevertheless, only 52% of vets routinely asked owners about behavioural changes during consultations. Forty-nine percent of respondents reported seeing pseudopregnancy in spayed bitches. The most commonly reported physical sign was enlarged mammary glands and/or milk production (89%). Treatment options varied (surgical, medical or none) and depended on duration and severity of physical and behavioural signs, owners’ preference, cost, concurrent disease, drug availability and previous history.

**Conclusions:**

This is the largest epidemiological study of canine pseudopregnancy in the UK. The prevalence and severity of clinical signs in dogs with pseudopregnancy are variable and possibly under-estimated. Dogs with overt pseudopregnancy experience diverse physical and behavioural changes and information on standard treatment protocols are lacking. Although, progress on our understanding of diagnosis and treatment of pseudopregnancy in spayed and entire bitches has been made, further studies are warranted.

## Background

Pseudopregnancy, also known as *pseudocyesis* is where an entire or spayed bitch shows clinical signs typical of the peri and post-partum period of pregnancy, despite the bitch not being pregnant. Pseudopregnancy can be further classified as overt, which is the clinical condition and covert, which is the normal physiological condition [1–12]. Pseudopregnancy in bitches has physiological and behavioural effects and is characterised by a range of physical and behavioural changes that commonly appear between six to eight weeks after oestrous [1, 2, 11–13]. Affected dogs may experience clinical signs including enlarged mammary glands and/or milk production, weight gain, vomiting, and appetite loss [1, 3–7, 13–17]. Behavioural signs consist of, but are not limited to, maternal behaviours including aggression in defence of resources, increased or reduced activity, nesting behaviour, and collecting or mothering objects [3–7, 13–18]. In some cases, the physical and behavioural signs can be marked.

The precise aetiology of clinical pseudopregnancy is still poorly understood, but is reported to be linked with the exposure and decline of plasma progesterone, high plasma prolactin concentrations, an increased tissue sensitivity to prolactin, or the existence of molecular variants of prolactin with varying bioactivities [3–6, 8, 11–14, 16, 19]. Based on clinical studies on pseudopregnancy in dogs, the estimated incidence of clinical pseudopregnancy can be as high as 50–75% in certain breeds (e.g. Afghan Hounds, Beagles, Boxers, and Dachshunds) [4, 5, 20].

Although pseudopregnancy is most commonly recognised in entire bitches, it can also develop as a result of spaying, particularly if susceptible bitches are spayed during dioestrous. Unless the relationship between spaying and the onset of clinical signs is very clear, veterinary surgeons may be less likely to recognise pseudopregnancy in a spayed bitch [3, 6, 10, 16]. It may be one cause of the increase in reactivity and/or aggression in bitches after being spayed [16, 21–23]. Aggression, in particular, is a potentially serious behavioural problem, which can result in not only injury to people, but also to dogs being relinquished or euthanised [18, 24, 25] and it is considered the most recurrent complaint in relation to canine behaviour. However, the extent of this as a direct result of pseudopregnancy is not yet known [10, 26–29].

There are currently no specific diagnostic tests for pseudopregnancy since hormonal assays are non-diagnostic for this condition [1, 10–12, 16]. Some dogs may present solely with behavioural signs, therefore a diagnosis of pseudopregnancy can be challenging.

Current epidemiological research literature and clinical trials pertaining to pseudopregnancy in the bitch is scarce, with only a handful of papers published since the late 1990s [13, 15, 16, 23, 30]. Therefore, a retrospective study was designed to advance our understanding of this complex condition. The questionnaire was designed to investigate through anamnesis, the prevalence, diagnosis, and treatment of canine pseudopregnancy in veterinary practices in the UK.

## Methods

The study was undertaken using a questionnaire-based postal study during a three-week period between October–November 2015. The questionnaire was sent to 2000 randomly selected veterinary surgeons that were registered on a market research database of veterinary surgeons (Vetfile), and members of the Royal College of Veterinary Surgeons in the UK in either small or mixed general practice. Referral practitioners, diplomate holders, locums, and veterinary surgeons in academia were excluded from the sample. The specific population sought was general practitioner vets, because they have a higher case load of pseudopregnancy and will be responsible for diagnosing the condition and making decisions about treatment. A prize draw for £300, postage paid return envelopes, and a summary of the survey results once analysed were offered as incentives for questionnaire completion and return.

### Questionnaire design

**Fig. 1:**
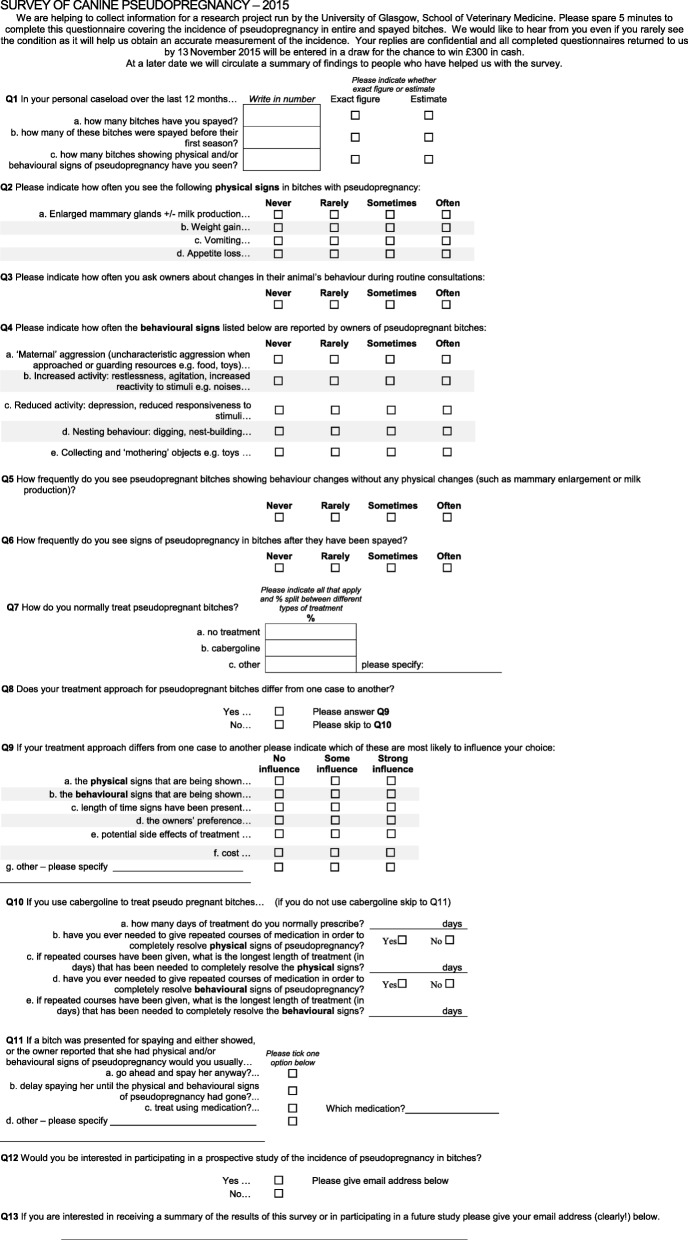
Canine pseudopregnancy postal questionnaire used for study

A copy of the questionnaire can be viewed in Fig. 1. Various question types, such as Likert-scale, dichotomous, closed-format, and open-format were used to obtain information about pseudopregnancy in canines. The survey included six statements associated with Likert-scale questions that reported on frequency (never, rarely, sometimes, often) and influence (no, some, strong). Additionally, a space for qualitative comments was available for the veterinary surgeons to expand on each of the open-ended questions. The raw data from the questionnaires was manually entered into a standard spreadsheet (comma separated values) file. There were three questions where either ‘exact’ or ‘estimated’ information could be given in regard to number of bitches spayed, bitches spayed before their first season, and bitches showing physical and/or behavioural signs of pseudopregnancy in the last 12 months. Supplementary comments on treatment approach were analysed thematically and appropriate larger categories created.

### Statistical analysis

Descriptive statistics were obtained using statistical analysis software (Analysis-Tool Kit for Microsoft Office Excel 2016) on the continuous data. The prevalence of the outcomes measured were estimated, specifically the prevalence of pseudopregnancy and clinical signs (physical or behavioural) commonly recognised as part of a diagnosis, and 95% confidence intervals (CI) were calculated [31]. Contingency table chi-square tests were used to assess associations between categorical variables or to identify differences in proportions with the level of significance set at *p* ≤ 0.05. To investigate the prevalence of canine pseudopregnancy, data were divided into categories. For example, the prevalence of pseudopregnancy was stratified by demographics (size, type, and structure of practice, job title and gender of veterinary surgeons, and region), physical signs, behavioural signs, and treatment used. Permission to conduct this study was granted by the University of Glasgow’s Research Ethics Committee.

## Results

### Demographics and descriptive analysis

All responses were collected during the three-week period of the survey. No reminders were sent out during that period. There was a response rate of 19.8% (397/2000) to the questionnaire. There were a small number of missing responses to various questions, ranging from 0 to 5% on any given questionnaire. The retrospective data was taken from the questionnaire responses, which was collected via anamnesis from the owner to veterinary surgeon.

A summary of respondent demographics is presented in Table 1. Thirty-nine percent of respondents (153/397) reported they would be interested in participating in a prospective study.

**Table 1:** Respondent demographics in postal questionnaire survey of pseudopregnancy in spayed and entire bitches

*Respondents*	Total	1–4 Vets	> 5 Vets	SA^a^ Practice	Mixed^b^ Practice	North & West^c^	South & East^d^	Vet Senior^e^	Vet Junior^f^	Private^g^	Corporate^h^	Charity
Sample Mailed	2000	960	1040	1380	620	1000	1000	700	1300	1605	336	59
		48%	52%	69%	31%	50%	50%	35%	65%	80%	17%	3%
Sample Analysed	397	196	201	278	118	188	209	113	284	331	60	8
		49%	51%	70%	30%	47%	53%	28%	72%	83%	15%	2%
Response Rate	19.8%											

^a^Small animal

^b^Small and large animals are seen in the practice

^c^Region including Scotland, Northern England, Wales, West Midlands, and Northern Ireland

^d^Region including East Anglia, East Midlands, London, South East England, and South West England

^e^Principal, partner, or buyer

^f^Assistant or regular locum

^g^Non-commercial veterinary practice that is independently owned

^h^Commercial veterinary practice or joint venture franchise

**Fig. 2:**
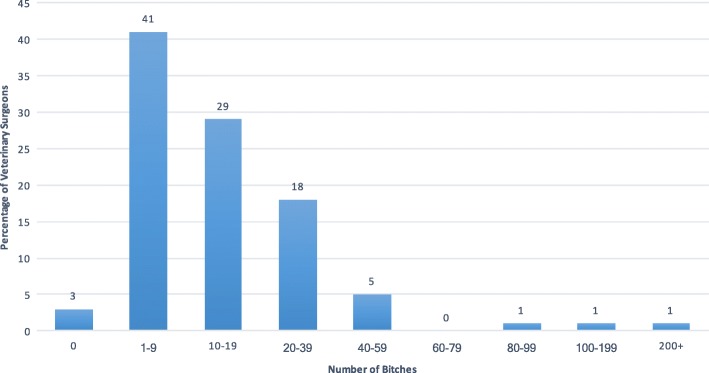
Number of bitches seen by veterinary surgeons during a 12-month period showing physical and/or behavioural signs of pseudopregnancy

Table 2 summarises the descriptive statistics. For the three questions (1a, b, and c), 84% of the answers were estimated figures rather than exact. Ninety-seven percent of veterinary surgeons had seen at least one case of pseudopregnancy in the previous 12 months (mean 16; median 10; range 0–250) (Fig. 2).

**Table 2:** Descriptive statistics on the number of bitches spayed, frequency of pseudopregnancy, and treatment length

	Number of bitches spayed in past 12 months	Number of bitches spayed in past 12 months before their first season	Number of bitches seen in past 12 months showing physical or behavioural signs of pseudopregnancy	Percentage of pseudopregnant bitches not treated	Percentage of pseudopregnant bitches treated with cabergoline	Percentage of pseudopregnant bitches treated with other treatments	Cabergoline: number of days treatment normally prescribed	Cabergoline: longest number of days needed to resolve physical signs	Cabergoline: longest number of days needed to resolve behavioural signs
Respondents	397	397	397	397	397	397	397	397	397
Missing	4	5	4	1	1	1	19	7	6
Sum	22,986	9744	6212	20,685	17,531	1207	2051	3299	849
Mean	58.5	24.9	15.8	52.2	44.3	3.0	5.7	13	13.3
Median	40	15	10	50	50	0	5	12	12
Standard Deviation	81.9	31.4	25.2	31.3	30	13.5	2.0	6.6	5.3
Range	1000	250	250	100	100	100	35	85	36
Minimum	0	0	0	0	0	0	1	5	6
Maximum	1000	250	250	100	100	100	36	90	42
IQR^a^	50	30	15	57.5	50	0	1	4	4
Confidence Level (95.0%)	8.1	3.1	2.5	3.1	3.0	1.4	0.2	0.8	1.3

^a^Interquartile range

Forty-two percent (9744/22,986) of bitches had been spayed by veterinary surgeons before their first season in the previous 12 months. Forty-nine percent of veterinary surgeons reported seeing pseudopregnancy in spayed bitches, however, of these, only 1% saw it often, 7% sometimes and 41% rarely.

### Physical signs and behavioural signs of pseudopregnancy

**Fig. 3:**
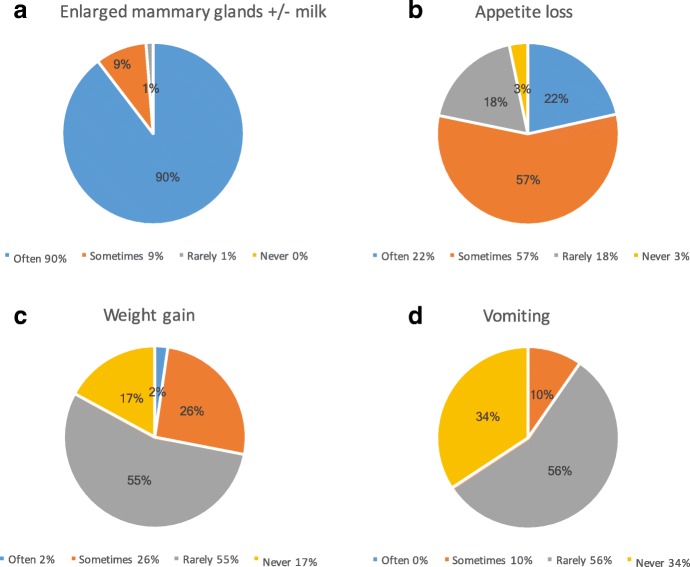
Frequency of reported clinical signs of pseudopregnancy in canines. **a** Enlarged mammary glands +/- milk. **b** Appetite loss. **c** Weight gain. **d** Vomiting

The most frequently reported clinical sign of pseudopregnancy was enlarged mammary glands and/or milk production (Fig. 3a), followed by appetite loss (Fig. 3b). Owners rarely reported weight gain (Fig. 3c) or vomiting (Fig. 3d).

**Fig. 4:**
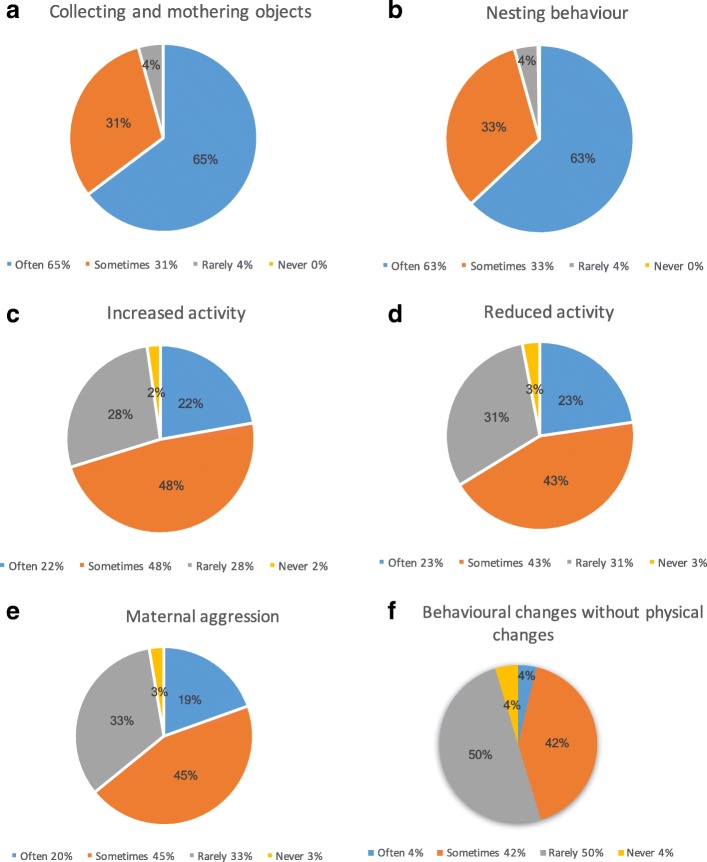
Frequency of reported behavioural signs of pseudopregnancy in canines. **a** Collecting and mothering objects. **b** Nesting behaviour. **c** Increased activity. **d** Reduced activity. **e** Maternal aggression. **f** Behavioural changes without physical changes

The behavioural sign most often seen was collecting and mothering objects (Fig. 4a) followed by nesting behaviour (Fig. 4b), increased activity (Fig. 4c), and reduced activity (Fig. 4d). Ninety-seven percent of vets indicated that they had seen maternal aggression in pseudopregnant bitches (Fig. 4e, 19% often; 44% sometimes; 33% rarely; 3% never).

Ninety-six percent of veterinary surgeons have seen behavioural changes, without any physical changes, as the main presenting sign for pseudopregnancy within the last 12 months. Of these, 4% reported this to be often, 41% sometimes and 50% rarely (Fig. 4f).

Only 52% of respondents reported that they often asked owners about behavioural changes during routine consultations (35% sometimes, 12% rarely and 1% never). Furthermore, senior vets (60%, 68/113) more frequently asked owners about changes in behaviour during routine consultations compared to junior vets (49%, 140/284).

### Treatment approach for pseudopregnancy

**Fig. 5:**
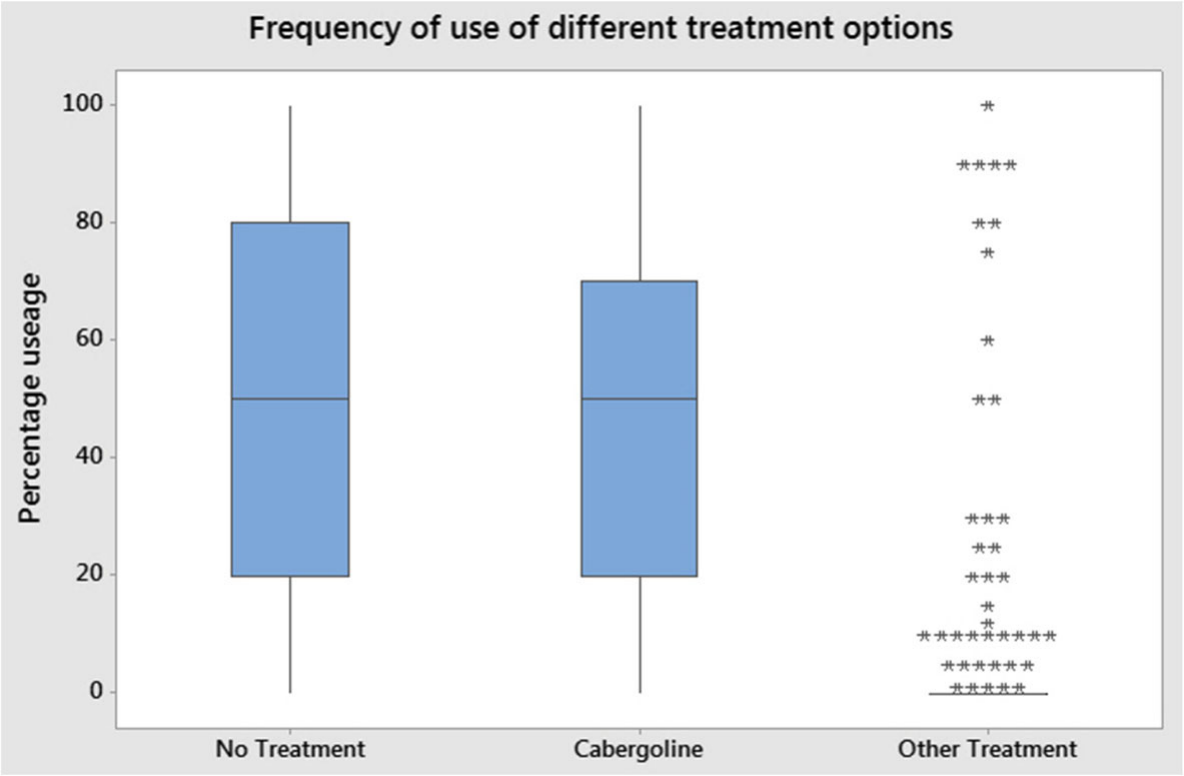
Frequency of reported use of different treatment approaches in pseudopregnant bitches. Other treatments used were Megestrol (Ovarid; Jurox), Proligestone (Delvosteron; Intervet), antibiotics, behavioural modification, diet, and exercise

Figure 5 shows the frequency of reported use of different treatment approaches by veterinary surgeons in pseudopregnant bitches. Mean number of responses for no treatment was 52% (median 50%), 44% for cabergoline (Galastop; Ceva, median 50%), and 3% for other treatment (median 0%). For the vets that used cabergoline, the median frequency for which they would use it was 50% of the time. Most of the responses were in the range of 20–80% for no treatment. The mean percentage of pseudopregnant bitches not treated was 52% (median 50%).

**Fig. 6:**
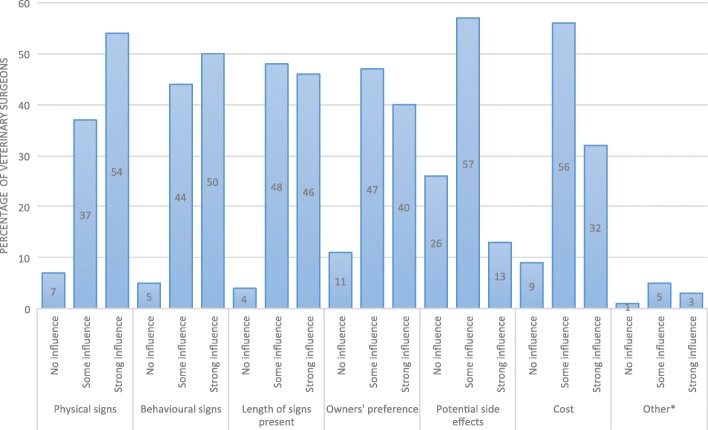
Influence on treatment choices in pseudopregnant bitches. *Other influence on treatment approach reported were concurrent disease, drug availability, spaying, and previous history of pseudopregnancy. There were some missing responses for this question and not every veterinary surgeon treated all pseudopregnant bitches

Eighty-eight percent of vets varied their treatment approach for pseudopregnant dogs, junior vets significantly more so than senior vets (*p* < 0.05). Physical signs, behavioural signs, and length of time signs had been present were most likely to influence the treatment choice (Fig. 6). In contrast, factors that only had some influence included owners’ preference, potential side effects and cost.

Typically, when bitches presented with pseudopregnancy they were either not treated (47%) or treated with cabergoline (48%). The remainder were treated with proligestone (Delvosteron; Intervet, 2%), megestrol (Ovarid; Jurox, 1%), and other (2%, behavioural modification, diet or exercise). Behavioural modification, diet, and exercise were described as the sole treatment approach by vets in charity practices.

The mean number of days that cabergoline was prescribed was 5.7 ± 0.1 (median 5). Furthermore, 18% of respondents reported that a repeated course of cabergoline was required to resolve behavioural signs and 68% respondents reported repeat courses were required for resolution of physical signs. The mean of longest number of days required for cabergoline to resolve both behavioural and physical signs was 13 days, although the range was bigger for resolution of physical signs (5–90 days compared to 6–42 days).

If a bitch presented for spaying and showed signs of pseudopregnancy, 96% of respondents would delay the procedure until the signs of overt pseudopregnancy had abated, whilst 4% of respondents would go ahead with the procedure regardless. Of those veterinary surgeons that would delay the procedure until physical and behavioural signs had resolved, 30% would treat medically and 4% would treat with behavioural modification. The most commonly reported medical treatment was cabergoline at 93%, followed by proligestone at 3%.

## Discussion

This epidemiological study is the largest and first to assess presenting physical and behavioural signs of canine pseudopregnancy and the subsequent treatment choices made by practising veterinary surgeons in the UK. The exact incidence of clinical pseudopregnancy is not known, but has been estimated to be between 50 and 75% [5] and 10–20% [14]. Mean prevalence of canine pseudopregnancy in the present study was 10 cases annually per vet and only 3% of veterinary surgeons did not encounter a case of pseudopregnancy in the previous year. If the survey population is representative of the total UK small animal practitioner population (10,022 on Vetfile database in June 2016), then approximately 100,000 cases of pseudopregnancy could occur annually (Personal communications with Market Research and Information Manager, Veterinary Business Development Ltd.).

Commonly measured reproductive hormones (prolactin and progesterone) clinically change in both the pregnant and overtly pseudopregnant bitch and cannot be reliably used as a diagnostic aid [1, 3, 4, 6, 10–13, 15]. Diagnosis of overt pseudopregnancy is usually based on timing of the onset of physical signs or behavioural changes in relation to the previous season in entire bitches, or to being spayed, followed by a positive response to treatment with a prolactin-reducing drug, such as cabergoline. Other differential diagnoses, such as true pregnancy or pyometra, should be excluded [3, 15].

Of the physical signs reported in this study, mammary gland enlargement and/or milk production were the most frequent. Findings in the current study accorded with those of Harvey et al. in that some pseudopregnant bitches do not show physical signs at all [14, 16, 17]. In this current study, 96% of vets saw cases of pseudopregnancy presenting with behavioural signs alone in the preceding 12 months. The two most common behavioural signs reported were collecting and mothering objects and nesting behaviour and this is the first study to have reported frequency of these signs. It is possible that a significant proportion of cases of pseudopregnancy are undiagnosed particularly as a significant number of vets in practice did not routinely ask about behavioural changes during consultations; this has been previously reported [24, 25].

Moreover, in an epidemiological study of behavioural problems, Fatjo and colleagues reported that more than 75% of veterinary surgeons estimated that at least 10% of euthanasias were related to behavioural problems [26] and are associated with an increased risk of relinquishment to rehoming centres [24]. The current survey indicates that aggression is not uncommonly seen in pseudopregnant bitches; this is pertinent since it has serious implications for both owners and dogs [9, 25, 29]. Since only 52% of respondents often asked owners about behavioural changes during routine consultations, it suggests that there is significant room for improvement, especially for junior vets, who are less likely to ask about behavioural changes. Modification of risk factors for pseudopregnancy (i.e. being sexually intact), by spaying or pharmacological interventions, could reduce its frequency in entire bitches and sequentially reduce behavioural problems in dogs.

Veterinary literature proposes that the best preventive method for pseudopregnancy is to spay the bitch before the onset of first oestrous [2, 5, 11, 12, 16, 17, 23], although there is some controversy over the behavioural and health-related effects of neutering before a season compared to after [32–36]. Otherwise, it is important to avoid spaying when a bitch has an overt pseudopregnancy or during the dioestral period [2, 5, 6, 11, 12, 14, 17, 30]. Spaying during the dioestral period results in a rapid decline in plasma progesterone and a rise in plasma prolactin concentration. Pseudopregnancy can then become overt and possibly persistent, especially in bitches with a history of pseudopregnancy before they were spayed [2, 6, 8, 10–12, 23, 30].

This study indicated that 4% of vets would go ahead and spay a dog even when showing signs of pseudopregnancy, thus it is important that veterinary surgeons are educated and advised against doing so. Ideally, the entire overtly pseudopregnant bitch should be treated medically and the surgery delayed until clinical signs subside, or serum progesterone levels are tested or risk the persistence of clinical signs of pseudopregnancy [6, 16, 23].

Since covert pseudopregnancy is a normal physiological condition, treatment is not required in many cases. However, treatment is warranted if physical or behavioural signs are extreme or last longer than 4 weeks, particularly if these are occurring in a bitch that has been spayed, otherwise signs might persist with each oestrous. Therefore, overt pseudopregnancy is a treatable condition with a good prognosis for resolution, as long as the underlying hormonal cause is recognised [10–12, 16, 37]. However, if under-recognised, as our data suggests, bitches may be treated inappropriately. Various classes of drugs have been specifically developed and used to treat pseudopregnancy, including anti-prolactins (Bromocriptine, Parlodel; Novartis, Cabergoline, Galastop; Ceva and Metergoline, Contralac; Virbac), progestogens (Proligestone, Delvosteron; Intervet and Megestrol, Ovarid; Jurox), serotonin agonists (Metergoline), and dopamine agonists (Bromocriptine and Cabergoline) [1, 2, 11, 12, 15, 37, 38]. Although, progestins were once widely used to treat overt pseudopregnancy in bitches, they are not fully effective because pseudopregnancy tends to recur once treatment is stopped and they have the potential to cause a wide range of serious side effects. Cabergoline is the suggested drug of choice for this condition, in part because it has the least side effects and longer duration of action [1, 11, 12, 15, 16, 23, 37, 39]. However, although safe, cabergoline is an expensive drug. It is a selective prolactin inhibitor and effective in suppressing prolactin release from the pituitary [1, 4, 23, 38]. It was the most commonly used drug in this study and 96% of vets reported prescribing it in the previous 12 months. The data showed cabergoline to be commonly prescribed for between five and 6 days. However, many vets in our study reported needing to use it for up to 13 days to resolve physical and behavioural signs of clinical pseudopregnancy. Ramsey states that cabergoline should be given for four to 6 days, but that control of aggression-related signs may require dosing for 14 days [38]. Bastan et al. used cabergoline in overtly pseudopregnant bitches and found that the enlarged mammary glands completely resolved by 7 days [1]. Some dogs (68%, 260/397) required repeated courses of cabergoline to resolve physical signs completely, which compares to Harvey and colleagues, who found the overall success rate of pseudopregnant bitches using cabergoline for 5 days was 73% (19/26) [23]. Cabergoline has been shown in many clinical studies to effectively reduce serum prolactin levels in 5 days [1, 16, 39–41]. Therefore, there should be improvement of clinical signs if pseudopregnancy is the correct diagnosis and there is not another cause of hyperprolactinaemia. As shown in this study, physical and behavioural signs took different lengths of time to resolve whether treatment was given or not. Of interest, was that up to 42 and 90 days of treatment were required for the behavioural and physical signs to disappear gradually, respectively. Whether physical signs are genuinely more difficult to resolve, or whether owners can ‘accept’ behavioural changes in their pets more readily than physical signs, or do not recognise the behavioural signs or other causes of clinical signs may be present, is open to debate and would require further investigation.

A minority of pseudopregnant bitches were treated by modification of behaviour, diet, and exercise. Specific behavioural management included, discouraging nesting and mothering behaviour, advising the owner to avoid touching or brushing the abdomen (i.e. specifically the mammary glands), and the application of an Elizabethan collar to reduce self-stimulation (e.g. licking) of the mammary glands, which could stimulate or perpetuate lactation. A range of factors were found to influence treatment choices, including physical and behavioural signs, duration of signs present, owners’ preference, cost, concurrent disease, drug availability, spaying, and previous history of pseudopregnancy. Treatment options varied amongst vets, especially between junior and senior vets (*p* < 0.05), which indicated that there is individual clinical assessment and treatment judgement was tailored for each pet. This is shown by the broad interquartile ranges in the treatment choices in Fig. 5.

It is important to note that 39% of the respondents indicated willingness to participate in a future prospective study; certainly, there are more questions to be answered, such as asking veterinary surgeons how often pseudopregnant spayed bitches showed signs of pseudopregnancy before they were spayed, investigating the risk factors for pseudopregnancy in entire and spayed bitches, breed predisposition to pseudopregnancy, and how veterinary surgeons are currently diagnosing pseudopregnancy. Further education about this common disorder in dogs would be valuable to ensure optimal diagnosis and treatment strategies. There is an urgent requirement for robustly designed clinical trials along with diagnostic information on pseudopregnancy, since few diagnostic indicators have been consistent across studies due to the lack of standardisation and agreement of hormonal measurements.

### Limitations and strengths of study

This study had a number of limitations that should be considered. This was a questionnaire-based study with a response rate of 19.8%, so numbers were limited. For a postal survey, the response rate is considered low [42], therefore an inherent respondent bias cannot be excluded due to the small sample size. We were unable to conduct an assessment of non-responder bias due to the vast majority of respondents being anonymous. General practitioner veterinary surgeons were chosen as participants in this study due to their expected higher case load of pseudopregnancy. However, there may have been different results from diplomates as they may have additional expertise in diagnosing and treating pseudopregnancy in bitches. As a retrospective study, some of the answers were estimated rather than exact, which may have incurred errors since they were not directly observed from the veterinary surgeon. In addition, some of the answers were semi-quantitative.

Diagnosis of pseudopregnancy was based on clinical signs characterised by physical and/or behavioural changes commonly seen in veterinary medicine [3–6, 11–17, 30]. Diagnosis is not clear cut, is often a diagnosis of exclusion, and false negative or false positive diagnoses may be made. A final limitation is the lack of diagnostic data in this study, however there is a current lack of standardisation and the validity of such data is difficult to compare.

A strength of this study was that the respondent selection was random and geographically diverse, which represented a large sampling of different practices within the UK. Valuable information on presentation, diagnosis and clinical signs was gleaned and adds to our knowledge on canine pseudopregnancy in the UK. Additionally, this research produced empirical data based on real observations from a wide variety of owners and veterinary surgeons in practice, enhancing our depth of knowledge on the topic of canine pseudopregnancy.

## Conclusions

This study was designed to evaluate different aspects of canine pseudopregnancy from the perspective of veterinary surgeons in general practice in the UK, since there is limited current data on the topic. Progress on our understanding of diagnosis and treatment of pseudopregnancy in spayed as well as entire bitches has been made. The main findings of the survey were that aggression is not uncommonly seen in pseudopregnant bitches, some covertly pseudopregnant bitches do not show obvious physical signs, pseudopregnancy can occur in spayed bitches, and not all vets routinely ask owners about behaviour during consultations. The prevalence and severity of clinical signs in dogs with pseudopregnancy in the UK seems to have been largely under-diagnosed in the past, since dogs with overt pseudopregnancy experience diverse physical and behavioural changes. The use of cabergoline has made a worldwide positive impact on the treatment of pseudopregnancy. Further, this study provided an estimate of the proportions of veterinary surgeons in the UK that see pseudopregnancy and common treatment protocols described. Clinicians should use their judgement for the most appropriate treatment plan for each patient. This study also highlights areas for future research; risk factors such as age, breed, parity, and environment have not been evaluated, frequency of post-spay pseudopregnancy in bitches, and how often this corresponds with aggressive behaviour also warrants investigation.

## Abbreviations

CI: Confidence intervals
UK: United Kingdom
